# Regulation of the Bud Dormancy Development and Release in Micropropagated Rhubarb ‘Malinowy’

**DOI:** 10.3390/ijms23031480

**Published:** 2022-01-27

**Authors:** Agnieszka Wojtania, Monika Markiewicz, Piotr Waligórski

**Affiliations:** 1 Department of Applied Biology, Institute of Horticulture—National Research Institute, Konstytucji 3 Maja 1/3 Street, 96-100 Skierniewice, Poland; monika.markiewicz@inhort.pl; 2 Department of Biotechnology, The *Franciszek Górski* Institute of Plant Physiology, Polish Academy of Sciences, Niezapominajek 21 Street, 30-239 Kraków, Poland; pewalig7@gmail.com

**Keywords:** endogenous hormone, gene expression, phenolics, soluble sugars, starch, temperature

## Abstract

Culinary rhubarb is a vegetable crop, valued for its stalks, very rich in different natural bioactive ingredients. In commercial rhubarb stalk production, the bud dormancy development and release are crucial processes that determine the yields and quality of stalks. To date, reports on rhubarb bud dormancy regulation, however, are lacking. It is known that dormancy status depends on cultivars. The study aimed to determine the dormancy regulation in a valuable selection of rhubarb ‘Malinowy’. Changes in carbohydrate, total phenolic, endogenous hormone levels, and gene expression levels during dormancy development and release were studied in micropropagated rhubarb plantlets. Dormancy developed at high temperature (25.5 °C), and long day. Leaf senescence and dying were consistent with a significant increase in starch, total phenolics, ABA, IAA and SA levels. Five weeks of cooling at 4 °C were sufficient to break dormancy, but rhizomes stored for a longer duration showed faster and more uniformity leaf growing, and higher stalk length. No growth response was observed for non-cooled rhizomes. The low temperature activated carbohydrate and hormone metabolism and signalling in the buds. The increased expression of *AMY3*, *BMY3*, *SUS3*, *BGLU17*, *GAMYB* genes were consistent with a decrease in starch and increase in soluble sugars levels during dormancy release. Moreover, some genes (*ZEP*, *ABF2*, *GASA4*, *GA2OX8*) related to ABA and GA metabolism and signal transduction were activated. The relationship between auxin (IAA, IBA, 5-Cl-IAA), and phenolic, including SA levels and dormancy status was also observed.

## 1. Introduction

Perennial herbaceous plants from temperate climate regions, such as rhubarb (*Rheum*; Polygonacea family), have an adaptive mechanism that alters active growth and bud dormancy that allows them to survive in adverse conditions during winter. These plants use cyclically changing environmental signals, such as temperature and day-length, to coordinate their growth and development with seasonal climate changes [1,2,3]. Thus, the proper timing of the onset and release of dormancy impact the survival, productivity, and spatial distribution [4]. The annual dormancy is classified into paradormancy, endodormancy, and ecodormancy based on the internal or external repression signals [5]. Studies on some woody plant species (*Prunus mume*, *Prunus avium*) [6,7], and several herbaceous perennials, such *Paeonia lactiflora* [8] and *Euphorbia esula* [9] have shown that dormancy induction and release are involved in numerous physiological changes, such as alteration in respiration rate, carbohydrate metabolism, growth regulator levels, water content, and the activities of other compounds. To date, reports on rhubarb in this area, however, are lacking.

Most *Rheum* species are native to the northern and central regions of Asia, where they have been cultivated for medical purposes for thousands of years [10]. Culinary rhubarb is popular as a vegetable crop, valued for its long, thickened leaf petioles (stalks) [11]. They contain high levels of organic acids (malic, citric, fumaric, and ascorbic acid), dietary fiber, protein, potassium, calcium, magnesium, and high levels of different phenolics, such as stilbenes, anthocyanins, and flavonols which have a range of bioactivities relevant to human health [12,13]. Rhubarb is grown commercially in the northern part of Europe, the USA, Canada, and Australia. In Poland, in the Subcarpathia region, rhubarb has been cultivated since the beginning of the 20th century, and the ‘Malinowy’ cultivar is the most popular. Consumers and the food industry prefer red stalks that are sweeter and have a higher content of polyphenolics [14]. The length, width, and colour of leaf stalks vary significantly among rhubarb cultivars [13].

It is known that the red-stalked cultivars and selections should be propagated vegetatively to maintain desirable traits. However, the production of rhubarb by division of crowns is limited by the low yield of mother plants (2–8 divisions per plant) and the risk of virus transfer. Tissue cultures are a useful tool for vegetative propagation of virus-free rhubarb plants and rapid multiplication of valuable genotypes [15,16,17]. Recently, an effective method of in vitro propagation of selected genotype of rhubarb ‘Malinowy’ was developed [18].

**Figure 1:**
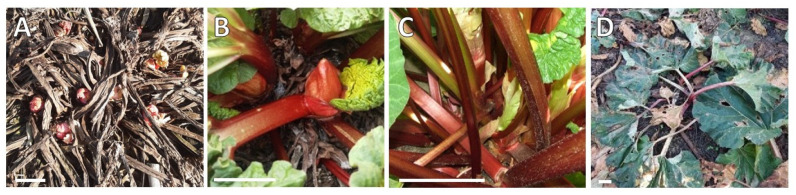
Annual growth cycle of rhubarb ‘Malinowy’ in the field; (**A**) 1 April, (**B**) 23 April, (**C**) 15 May, (**D**) 10 October.

In commercial rhubarb stalk production, either under the field or forcing cultivation, the bud dormancy development and release are crucial processes that determine the yields and quantity of stalks [19]. It is known that rhubarb cultivars vary in their depth of dormancy. However, this cultivar characteristic is not sufficiently understood. In the Northern Hemisphere, bud sprouting is observed in early Spring (March). Most rhubarb cultivars reach full maturity within two months of the growing season. When temperature declines in autumn (October), the process of the rhubarb leaf senescence starts, and the underground buds enter dormancy (Figure 1). After the cold winter period passes, bud sprouting is observed. It takes six to seven months under natural conditions to break dormancy.

On the other hand, during in vitro propagation of rhubarb, dormancy has not been developed. Shoots growing on MS medium containing growth regulators stay active around the year [18]. As it was shown, after in vitro rooting, the shoots were successfully acclimatized to ex vitro conditions. After planting to the field (in May), the plantlets of rhubarb ‘Malinowy’ become dormant, and then activate the growth after winter chilling (data not shown). So far, there is no information on bud dormancy regulation of rhubarb plantlets propagated in vitro.

The study aimed to determine the dormancy regulation in the valuable selection of rhubarb ‘Malinowy’ characterized by a high content of anthocyanins—cyanidin-3-O-rutinoside and cyanidin-3-O-glucoside. Changes in carbohydrate, total phenolic, endogenous hormone levels, and expression levels of related genes during dormancy development and release were studied in micropropagated rhubarb plantlets.

## 2. Results

### 2.1. Growth of In Vitro Propagated Plantlets in the Greenhouse and Dormancy Induction

**Figure 2:**
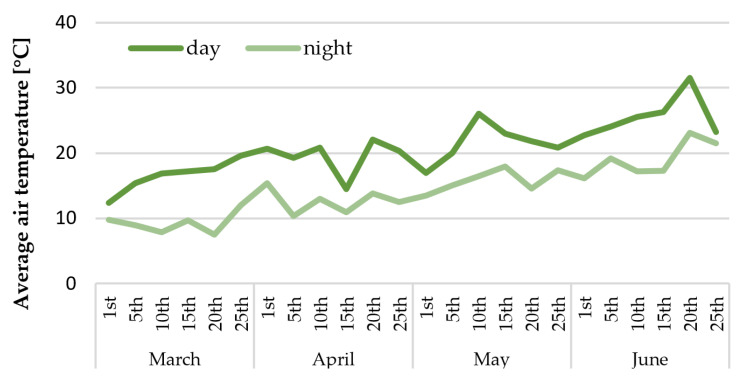
Temperature change during rhubarb growth in the greenhouse.

**Figure 3:**
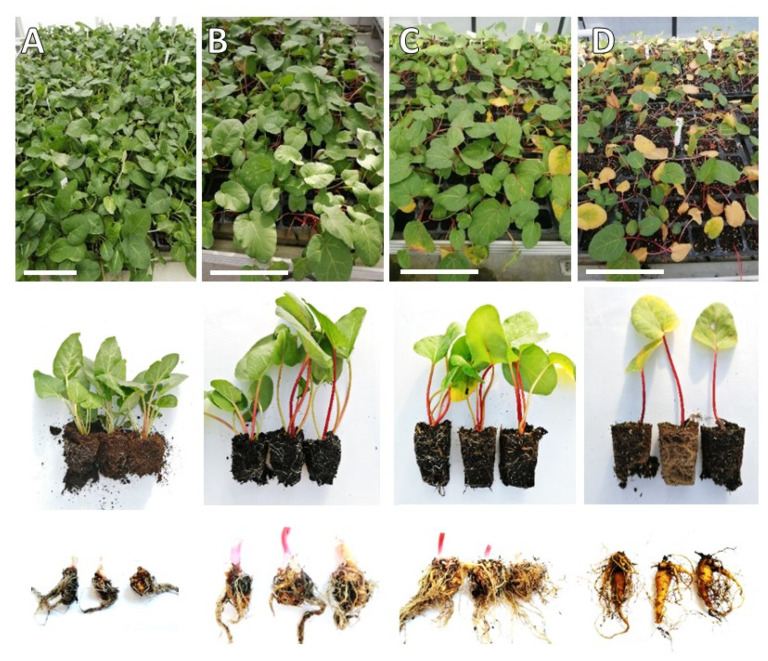
Plantlets of rhubarb ‘Malinowy’ propagated in vitro after different growth periods (**A**—1 month, **B**—2 months, **C**—3 months, **D**—4 months) in the greenhouse.

After the acclimatization in a growth room, the in vitro propagated plantlets of culinary rhubarb ‘Malinowy’ were transferred to the greenhouse (early March) to determine the growth characteristic of planting material. The monthly air temperature changes for the period March-June are shown in Figure 2. It has been observed that after the short adaptation period (approximately one–two weeks) to the greenhouse conditions, the rhubarb plantlets showed intensive growth of leaves and rhizomes. Maximum petiole length and leaf area were observed after two months of growth in the greenhouse (Table 1). After this period, the pace of growth was slowing down, and yellowing of the oldest leaves was observed (Figure 3). On the other hand, the mass of rhizomes was increasing. Over the fourth month of growth in the greenhouse, a rapid increase in leaf senescence has been observed. At the end of the fourth month approx. 50% of plantlets were leafless (Table 1; Figure 3).

### 2.2. Growth of In Vitro Propagated Plantlets in the Greenhouse and Dormancy Induction

To characterise the physiological changes that occur during four months of the rhubarb ‘Malinowy’ growth in the greenhouse, the content of soluble sugars, starch, phenolic and endogenous hormones in the buds was determined.

#### 2.2.1. Changes in the Carbohydrates and Total Phenolic Contents

**Figure 4:**
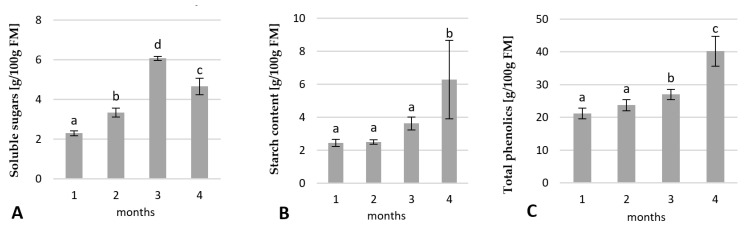
Variation in the content of soluble sugars (**A**), starch (**B**), and total phenolics (**C**) in the rhubarb buds during four months of growth in the greenhouse after in vitro conditions. Means indicated with the same letter within each growth parameter do not differ significantly (*p* = 0.05) according to Duncan’s test.

The contents of soluble sugars, starch, and total phenolics in the rhubarb buds were analysed after 1, 2, 3 and 4 months of growing in the greenhouse. All tested parameters were the lowest at the beginning of the growth in the greenhouse (Figure 4). The contents of soluble sugars increased 2.6-fold over the three months of growth in the greenhouse but decreased rapidly over the next month by 25%. The starch content in the rhubarb buds remained constant to the end of the second month, then started to increase. The highest increase of starch was observed over the fourth month of the growth in the greenhouse. The levels of phenolics in the buds also increased slowly over three months, then enhanced rapidly over the fourth month (Figure 4C). During four months of growth in the greenhouse, levels of starch and total phenolics were enhanced by 62% and 48%, respectively (Figure 4B). The high starch levels and total phenolics in the rhubarb buds coincided with rapid leaf yellowing of the rhubarb plantlets.

#### 2.2.2. Change in the Phytohormone Contents

**Figure 5:**
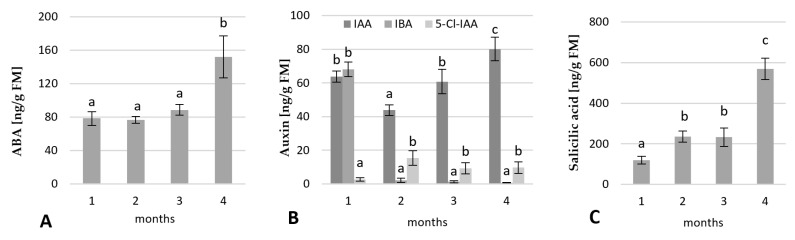
Variation in the content of ABA (**A**), auxins (**B**); IAA—indole-3-acetic acid, IBA—indole-3-butyric acid, 5-Cl-IAA—5-chloroindole-3-acetic acid, and salicylic acid (**C**) in the rhubarb buds during four months of the growth in the greenhouse after in vitro conditions. Means indicated with the same letter within each phytohormone and auxin type do not differ significantly (*p* = 0.05) according to Duncan’s test.

The contents of endogenous hormones in the rhubarb buds were also analysed after 1, 2, 3 and 4 months of cultivation in the greenhouse. The abscisic acid (ABA) contents remained essentially unchanged to the end of the third month, then increased by 50% over the fourth month of growth in the greenhouse (Figure 5). As shown in Figure 5B, rhubarb buds contained three types of auxin. The most abundant was indole-3-acetic acid (IAA), followed by indole-3-butyric acid (IBA), and 5-chloroindole-3-acetic acid (5-Cl-IAA). Their levels changed during the growth of plantlets in the greenhouse. At the beginning, the buds were characterized by a high content of both IAA and IBA. However, the level of IBA decreased drastically (by 96.3%) over the second month of the growth in the greenhouse, and a low level remained throughout all transition stages. The content of IAA in the buds also lowered over second month of the growing in the greenhouse, but only by 31.1%, and then started to increase to reach maximum value at the end of the fourth month (Figure 5B). The content of 5-Cl-IAA first increased over the second month, then decreased and remained constant to the end of cultivation. However, the levels of 5-Cl-IAA were almost significantly lower than the IAA. For example, dormant buds contained IAA and 5-Cl-IAA at a ratio of 9:1. These results demonstrated that a high level of IAA in the buds was consistent with a rapid senescence of rhubarb plantlets. It might suggest that auxin is involved in the development of rhubarb bud dormancy.

The content of salicylic acid (SA) in the rhubarb buds increased slowly over three months, then enhanced rapidly over the fourth month (Figure 5C). During four months of growth in the greenhouse, levels of SA were enhanced by 80%.

### 2.3. Break of Dormancy/Chilling Requirements for Shoot Induction and Growth

**Figure 6:**
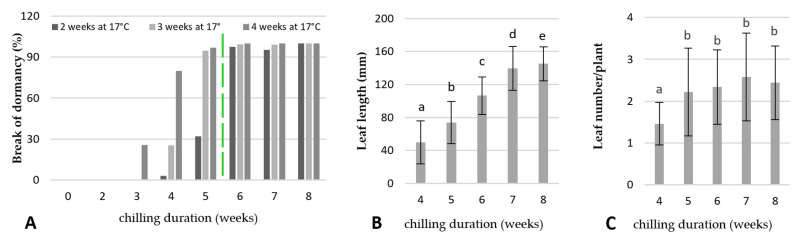
Effect of chilling duration (0, 2, 4, 5, 6, 7, 8 weeks) at 4 °C on a break of dormancy of rhubarb buds (**A**). The sprouting percentage was determined after two, three, and four weeks of growing at 17 °C following cold treatment. The green dashed line suggests the time of bud transition from endo- to ecodormancy. Leaf length and leaf number were determined after four weeks of cold treatment (**B**,**C**). Means indicated with the same letter within each growth parameter do not differ significantly (*p* = 0.05) according to Duncan’s test.

As shown in Figure 6A, cold treatment (4 °C) duration had a strong effect on breaking the dormancy of the rhubarb ‘Malinowy’. All of the control rhizomes (non-cooled) failed to sprout under growing conditions of 17 °C in a growing room until the end of the experiment and the next 12 months (data not shown). No sprouting response was also observed for rhizomes cooled for two weeks. Five weeks of cooling were sufficient to break dormancy (96.9%) in four weeks. However, rhizomes stored for a longer duration at 4 °C showed faster and more uniform leaf sprouting (Figure 6A). Rhizomes cooled for six, seven, and eight weeks sprouted 97.5%, 95%, and 100%, respectively, within two weeks. However, the rhizomes cooled for eight weeks produced the longest leaf petioles after four weeks of cultivation at 17 °C (Figure 6B). On the other hand, an increasing cooling period from five weeks to eight weeks had no influence on the number of formed leaves (Figure 6C).

### 2.4. Physiological Responses of the Underground Buds during Cold Treatment

For the determination of physiological indices related to rhubarb ‘Malinowy’ dormancy breaking, after each chilling duration, the content of soluble sugars, starch, total phenolics, and endogenous hormone were analysed.

#### 2.4.1. Changes in the Carbohydrates and Total Phenolic Contents

**Figure 7:**
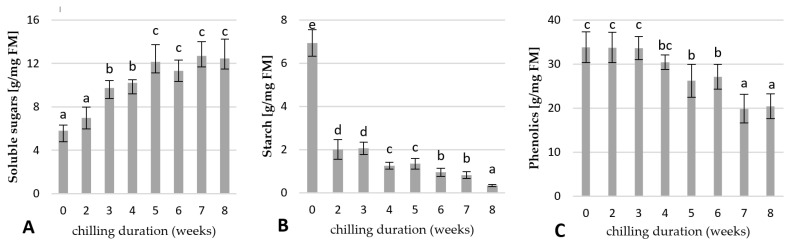
Variation in soluble sugars (**A**), starch (**B**), and total phenolic contents (**C**) in rhubarb buds during storage at 4 °C after different durations (0, 2, 3, 4, 5, 6, 7, and 8 weeks). Means indicated with the same letter within each growth parameter do not differ significantly (*p* = 0.05) according to Duncan’s test.

The contents of soluble sugars, starch, and total phenolics in the rhubarb buds were analysed after 0, 2, 3, 4, 5, 6, 7, and 8 weeks of the storage of the rhizomes at 4 °C. As shown in Figure 7, the buds of uncooled rhubarb rhizomes were characterized by a high level of starch and total phenolics, and low levels of soluble sugars. Exposure of rhizomes to a low temperature resulted in a decrease of starch and phenolic compounds and the accumulation of soluble sugars in the buds. Starch content in the rhubarb buds was inversely proportional to cooling duration. A significant decrease of starch content (by 3.4 times) was observed over two weeks of cold treatment. The lowest starch level was found in the buds after eight weeks of cooling (Figure 7B). The content of the soluble sugars was the lowest in the non-cooled rhubarb buds. Increasing chilling duration resulted in a significant increase of the soluble sugars over five weeks of storage at 4 °C. Then, the soluble sugar contents remained essentially unchanged to the end of the eight weeks (Figure 7A). High levels of soluble sugars in the buds coincided with dormancy release (Figure 6A and Figure 7A).

As shown in Figure 7C, the amount of total phenolics in the rhubarb buds showed no changes over three weeks of the rhizomes cooling at 4 °C. Then, their levels decreased by 40% with increasing cooling duration to seven weeks. Decreased phenolic levels coincided with increased bud sprouting (Figure 6A and Figure 7C).

#### 2.4.2. Change in the Endogenous Hormone Contents

**Figure 8:**
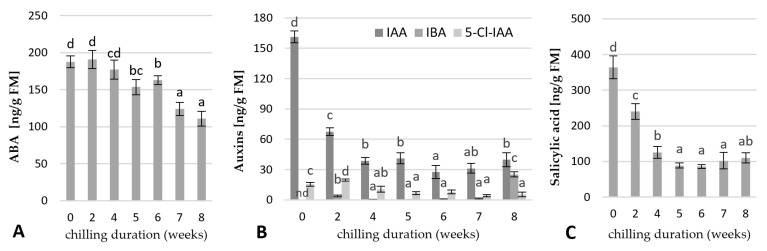
Variation in the content of ABA (**A**), auxins (**B**); IAA—indole-3-acetic acid, IBA—indole-3-butyric acid, 5-Cl-IAA—5-chloroindole-3-acetic acid, and salicylic acid (**C**) in rhubarb buds during storage at 4 °C after different durations (0, 2, 4, 5, 6, 7, and 8 weeks). Means indicated with the same letter within each phytohormone and auxin type do not differ significantly (*p* = 0.05) according to Duncan’s test.

The contents of endogenous hormones in the rhubarb buds were analysed after 0, 2, 4, 5, 6, 7, and 8 weeks of the storage of the rhizomes at 4 °C. As shown in Figure 8, the buds of uncooled rhubarb rhizomes are characterized by high levels of ABA, IAA, and SA. Exposure of rhizomes to low temperatures resulted in a decrease of all parameters. The ABA contents remained essentially unchanged for two weeks of cooling, then started to decrease reaching the lowest value after seven weeks of cold treatments (Figure 8A). The uncooled buds were characterized by a very high level of IAA. Its levels decreased drastically (by 83%) until week five of cooling, reached their lowest level after week six and then began to increase to the end of cooling treatment. Extending cooling to eight weeks resulted in a 50-fold increase of IBA levels in the rhubarb buds. The content of 5-Cl-IAA was the highest at the beginning of cooling treatment, then began to decrease from the fourth to the sixth week of cooling and remained constant to the end of cooling duration (Figure 8B). As shown in Figure 5C, the levels of SA in the rhubarb buds decreased by 76% over five weeks of cooling, then remained unchanged to the end of cooling treatments.

### 2.5. Expression Analysis of Dormancy-Related Genes in Plants during Cold Treatment

#### 2.5.1. Expression of Genes Related to Carbohydrate and Flavonoid Metabolism

We examined the expression of genes related to carbohydrate and flavonoid metabolism to determine whether changes in gene expression are linked to a shift in carbohydrate and phenolic compound levels during rhubarb dormancy release. The gene expression was analysed after 0, 2, 3, 4, 5, 6, 7, and 8 weeks of the storage of rhizomes at 4 °C.

**Figure 9:**
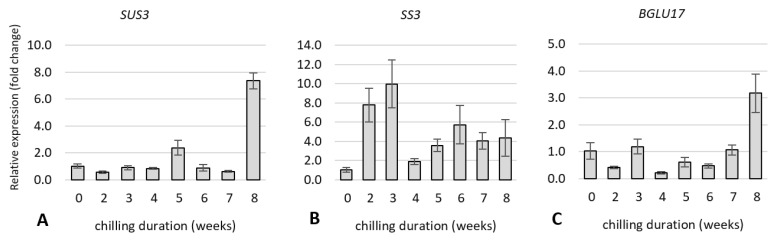

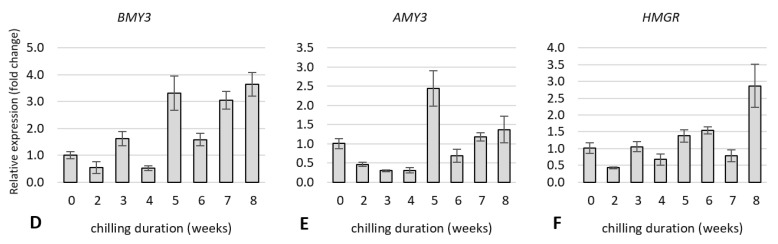
The relative expression of the genes involved in carbohydrate metabolism (**A**–**E**) and flavonoids (**F**) in rhubarb ‘Malinowy’ plants during cold treatment.

As shown in Figure 9, the levels of relative expression of *sucrose synthase 3* (*SUS3*) and *β-glucosidase* (*BGLU17*) were the highest in the buds eight weeks after cold treatment and were almost 8.0-fold and 3.0-fold higher, respectively, as compared to buds taken from non-cooled rhizomes. Among the starch amylases gene examined, *α-amylase* (*AMY3*) were drastically upregulated at a five-week cooling period. However, *β-amylase 3* (*BMY3*) was slightly upregulated after three weeks and then increased significantly, reaching the maximum value after five and eight weeks, 3.3-fold and 3.6-fold higher, respectively, as compared to the control. The *starch synthase 3* (*SS3*) was drastically up-regulated after two, and three weeks of cool storage, then up-regulated slightly from five to eight weeks of cool treatments. The gene of *3-hydroxy-3-methylglutaryl-coenzyme A reductase 1* (*HMGR*) related to phenolic metabolism, reached the maximum value after eight weeks of cold treatment, which was 3.0-fold higher than the control.

#### 2.5.2. Expression of Genes Related to Hormone Metabolism and Hormone Signal Transduction Pathway

We examined the expression of genes related to ABA and GA metabolism and hormone signal transduction to determine whether changes in gene expression are linked to shift in endogenous ABA levels during bud dormancy release in rhubarb. The gene expression was analysed after 0, 2, 3, 4, 5, 6, 7, and 8 weeks of the storage of rhizomes at 4 °C.

**Figure 10:**
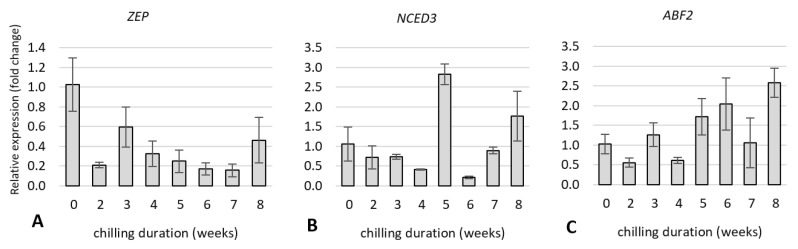

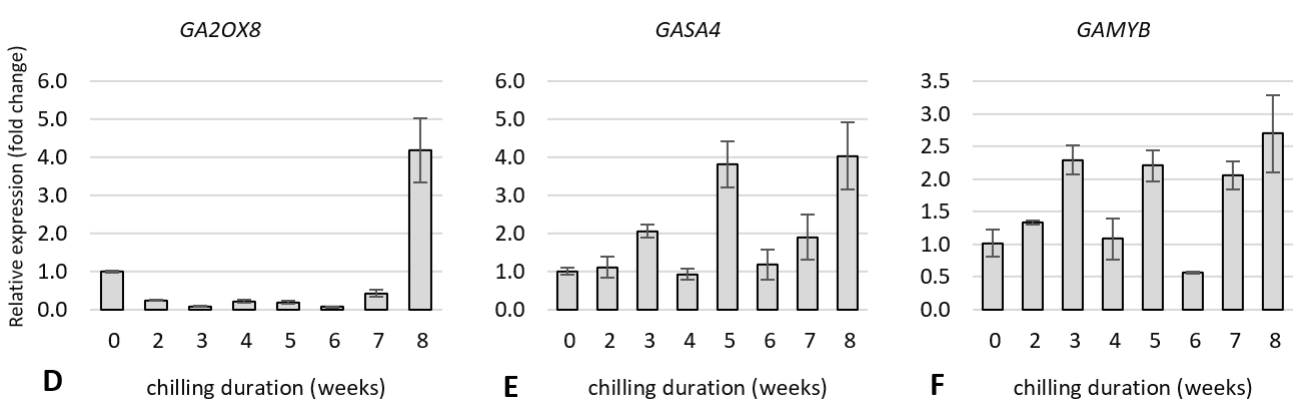
The relative expression of the genes involved in plant hormone metabolism, ABA (**A**–**C**) and GA (**D**–**F**) in rhubarb ‘Malinowy’ plants during cold treatment.

Among the genes related to ABA metabolism, *9-cis-epoxycarotenoid dioxygenase 3* (*NCED3*), and *abscisic acid responsive elements-binding factor 2* (*ABF2*) showed an increased level of relative expression compared to control. In cooled plants, the increase of *ABF2* transcripts level was observed up to the three weeks and reached the maximum value after eight weeks of cold treatment. The highest level of relative expression of *NCED3* was observed in plants after five weeks of cold treatment and was almost 3.0-fold higher compared to control. *Zeaxanthin epoxidase* (*ZEP*) gene related to ABA biosynthesis was down-regulated for all cooling treatments (Figure 10A–C).

The expression of genes related to GA synthesis—*GAST1 protein homolog 4* (*GASA4*) and *gibberellin 2-oxidase 8* (*GA2OX8*) increased in rhubarb buds with cooling duration in time. The relative expression level of these genes peaked at eight weeks of cold treatment and was 4.0-fold higher than control. Additionally, for *gibberellin-dependent* *α-amylase* (*GAMYB*), the level of relative expression was the highest after eight weeks of cold treatment and reached the value 2.7-fold higher compared to control. The increased expression level of this gene was also observed after three, five and seven weeks of cold treatment (Figure 10D–F).

## 3. Discussion

### 3.1. Dormancy Induction

In the literature, there is no information on bud dormancy induction and release in planting material of rhubarb propagated in vitro. Kozak and Sałata [15] reported the survival rate and growth of in vitro propagated rhubarb ‘Karpov Lipskiego’, first after one month of growth in the greenhouse and then after eight months of the growth in the field, following overwintering. For *Rheum rhabarbarum*, Clapa et al. [16] mentioned the formation of rhizomes after six months of growing ex vitro. The present work has shown, that the growth of rhubarb ‘Malinowy’ plantlets stopped over the third month of growth in the greenhouse at an average temperature of 21.4 °C and 16 h photoperiod, and then rapid leaf senescence was observed over the fourth month at an average air temperature of 25.5 °C (max. 28.1 °C). Growth cessation may suggest induction of paradormancy in rhubarb plantlets, however, yellowing and dying of leaves may indicate transition from para- to endodormancy. A similar result was reported in raspberry, where deeper dormancy was induced at 20 °C than at 4 °C [20]. So far, little is known about the role of high temperature in the induction of endodormancy. Generally, high temperature is considered a critical factor determining the growth pattern of rhubarb plants in the field. The yield and quality of rhubarb decrease when average summer temperatures rise above 27–32.2 °C, but the plants usually develop endodormancy in the autumn when the temperature decrease [19]. To reveal if high temperature is the main factor inducing the dormancy in rhubarb plantlets, further study is needed.

It is known that changes in carbohydrate dynamics are linked to changes in dormancy status [21,22,23]. Similar to results presented for leafy spurge [24], the inhibition of rhubarb petiole growth coincided with high carbohydrate levels in the buds. This suggests a high metabolic activity of rhubarb buds during paradormancy. In leafy spurge, it has been demonstrated that high sugar levels inhibited growth by antagonizing the perception of GA and increasing ABA perception [25,26]. Soluble sugars were shown to decrease in the buds with endodormancy development, and starch accumulation increased [27]. Similarly, we observed a clear relationship between the leaf senescence of rhubarb plantlets and the accumulation of starch in the buds. Starch is the most common storage form of carbohydrates in trees, and herbaceous perennials, that act as reserve molecules in order to support future growth [3,28].

The endogenous hormone contents and balance are important factors regulating a bud dormancy induction [29]. Abscisic acid (ABA) is considered a key endogenous inductor of dormancy. As shown in our study, ABA levels significantly were enhanced in the micropropagated rhubarb plantlets over the fourth month of growing in a greenhouse. An increase in the ABA levels during dormancy induction has been previously reported for different plant species, including *Vitis vinifera*, *Prunus persica*, *Pyrus pyrifolia*, and *Paeonia lactiflora* [8,29,30,31]. Under artificial stimuli, the depth of dormancy significantly depended on ABA concentrations [29]. Additionally, crosstalk was reported between ABA and sugars. It is known that ABA inhibits the activity of α-amylases, the key enzymes in starch degradation. Similar to the study on *Vitis vinifera* [32], we observed a simultaneous increase in ABA and starch levels in the rhubarb buds.

In contrast to ABA, the role of auxin in the bud dormancy is less known. Similar to our study, enhanced levels of endogenous IAA were found in dormant buds of *Paeonia lactiflora* [8] and *Solanum tuberosum* [33]. However, many reports are showing the opposite trend, for example, in *Euphorbia esula* [9], *Betula pendula* [34], and *Polianthes tuberosa* [35]. Recent studies have indicated that auxin-mediated seed dormancy is dependent on ABA [36] and is involved in the flavonol biosynthetic process [37].

In the present study, significant enhancement of total phenolics level and SA suggested that also phenolics might participate in the rhubarb bud dormancy development. Phenolics are known to be a very diverse group of secondary metabolites varying in composition and amount between genotypes, tissues, developmental stage, and environmental conditions [38]. The phenylpropanoid biosynthetic pathways can be induced by various stresses, low availability of nutrients, increased C/N ratio, and various growth regulators, including auxins, cytokinins, and jasmonic acid [38]. Phenolics protect plants from oxidative stress and are involved in the regulation of organogenesis and dormancy [39,40]. Salicylic acid (SA), a phenolic compound, has been the focus of intensive research due to its function as an endogenous signal mediating response to major biotic and abiotic stresses [41]. Xie et al. [42] found that SA inhibited α-amylase production by decreasing a GA-mediated α-amylase expression during rice germination. So far, little is known about the role of SA in bud dormancy.

### 3.2. Dormancy Release

Low temperature is the primary environmental signal regulating dormancy release in different plant species [21,43,44,45]. It has been reported that rhubarb requires at least 500 h of winter temperatures between 2.2 °C and 9.4 °C to break dormancy [46]. It is known that cold requirements do vary significantly among rhubarb cultivars, but their characteristics are not sufficiently understood. This study indicates that rhizomes of rhubarb ‘Malinowy’ treated at 4 °C for eight weeks showed the largest sprouting rate in the shorter time period and produce the longest stalks than those cooled for five to seven weeks. A positive relationship between cooling duration and sprouting has been previously reported for different plant species, including *Paeonia lactiflora* [47] and *Lilium* [45,48].

**Figure 11:**
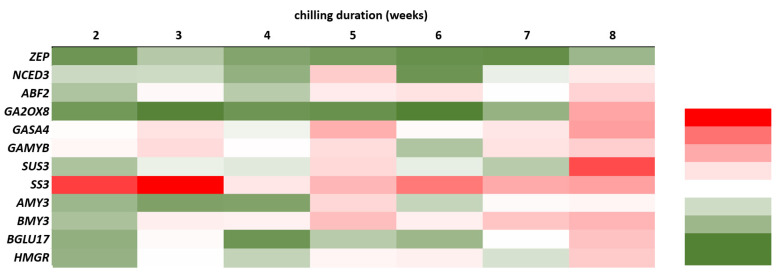
Heat map representation of relative dormancy-related genes expression. Samples are represented in columns, whereas genes are shown in rows. The differences in expression level are shown in distinct colors. Red indicates high relative gene expression and green indicates low relative gene expression.

Carbohydrate availability is considered to be of major relevance to the control of bud growth [27]. Many reports have shown that bud dormancy release is related to a decrease of starch levels and an increase of soluble sugars in the buds [6,8,45,48]. It is known that soluble sugars, mainly sucrose, could promote bud dormancy release, not only as an energy supply but also as a signalling molecule [26,49]. This study showed that low-temperature treatment was sufficient to activate carbohydrate metabolism in rhubarb buds. The increased expression of *α-amylase* (*AMY3*) and *β-amylase 3* (*BMY3*) genes were consistent with a decrease in starch during dormancy release. An increased expression of *sucrose synthase 3* (*SUS3*) in the rhubarb buds at the end of the cooling period might suggest that an earlier increase of soluble sugar content was related to starch catabolism than *de novo* sucrose synthesis. With the dormancy release process in rhubarb buds, up-regulation of *BGLU17* involved in sugar transport was also observed (Figure 11). Similar results have been reported in other plant species, including *Paeonia lactiflora*, *Lilium*, and *Prunus mume* [6,8,45,48]. In rhubarb, a little surprising was a significant up-regulation of *starch synthase 3* (*SS3*) for two weeks of chilling duration that was not consistent with the starch content in buds.

It is known that sugar alone, or through interaction with hormones, can induce or suppress many growth-related genes [49]. Additionally, crosstalk has been reported between sugar and hormones, including ABA, GA, IAA, and SA [26,50]. It has been demonstrated that α-amylases, key enzymes in starch degradation, playing a critical role in dormancy release are negatively regulated by ABA and positively by GA [32]. The enhanced expression of *GAMYB* in rhubarb buds during dormancy release might suggest the GA-promoted production of α-amylases. Generally, the balance between ABA and GA significantly influences dormancy regulation [51,52,53]. These results indicated that the decreased expression of ABA precursor (*ZEP*) and increased levels of *ABF2* involved in ABA signal transduction and metabolism were consistent with the reduced levels of ABA in the rhubarb buds during dormancy release. The opposite trend was observed in the expression of genes involved in gibberellin synthesis and metabolism (*GASA4*, *GA2OX8*) (Figure 11). Our results are in agreement with the response of *Paeonia lactiflora* [8] and *Prunus mume* [54]. Trangenic studies showed that ABA and GA are involved in the metabolic regulation of each other [55], and GA responses can be inhibited by high levels of ABA [56].

Additionally, this research showed significant changes in auxin concentrations and types during bud dormancy release. The most abundant was IAA, the main active auxin in different plant species, followed by IBA (auxin precursor), and chlorinated auxin 5-Cl-IAA, known to be more active than IAA, but found only in certain higher plant species [57,58]. Being a major growth promotor, IAA has been implicated in dormancy release in many plant species. However, in some plant species, including silver birch and tea plants [34,59], the IAA levels remain low during the dormancy induction and increase steadily after dormancy release until bud break. These results are in agreement with those obtained for *Solanum tuberosum* [33], where deep dormancy correlated with high auxin levels. Then its content first decreased during cooling treatment and then started to increase when dormancy was released. In rhubarb, extending rhizome cooling to eight weeks resulted in a significant increase both of IAA, and IBA levels in the buds.

Data in the literature also indicate a link between the changes of phenolic levels and dormancy release. However, the relationship between phenolic levels and dormancy release has remained controversial, due to the differences in temperature and chilling duration. For example, in grapevine [60,61] and pistachio [62], the total phenolic levels, including SA enhanced during dormancy release. It was explained as a defensive mechanism to improve tolerance and adaptability to low temperatures. Our study is in agreement with those for different kiwi cultivars [63] and onion [64], that phenolics levels decreased with cooling duration. Those results indicated that the reduced levels of phenolics in the rhubarb buds were consistent with the enhanced expression *HMGR* gene related to phenolic metabolism (Figure 11).

## 4. Materials and Methods

### 4.1. Plant Material

Micropropagated planting material of selected genotype of rhubarb ‘Malinowy’ characterized by a high content of anthocyanins-cyanidin-3-O-rutinoside and cyanidin-3-O-glucoside was used for the study. In vitro shoot cultures were established and multiplicated by axillary shoot growth stimulation [18]. Then, the in vitro rooted shoots were planted in multicellular trays of 33 mm diameter with a mixture of peat (Alonet, Skierniewice, Poland) and perlite (2:1), in plastic plug boxes covered with transparent plastic caps to prevent dehydration. Plantlets were grown in a growth room (25 ± 2 °C; PPFD—50 µmol m^−2^s^−1^). The plantlets were hardened by gradually decreased air humidity. After ten days, they were fed with 0.1% Kristalon (Yara, Oslo, Norway) containing 18:18:18 (*w*/*w*/*w*) NPK.

### 4.2. Growth in the Greenhouse/Dormancy Induction

After four weeks of acclimatization, the rhubarb planting material was transferred to a greenhouse in early March 2021. They were grown at natural temperature and 16 h photoperiod provided by additional lighting (120 µmol m^−2^ s^−1^). Plants were manually watered and fed with 0.1% Kristalon every two weeks. After 1, 2, 3, 4 months, plantlets growth (length of leaf petioles, leaf area, rhizome mass) and quality (yellow leaves) were determined. Every month, twenty buds were selected randomly and pooled to determine the contents of soluble sugars, starch, total phenolics and endogenous hormone.

### 4.3. Evaluation of Dormancy Breaking in Buds at Controlled Conditions

After leaf-fall (four months after the transfer to the greenhouse), the rhubarb ‘Malinowy’ dormant rhizomes were stored at 4 °C without light for different cooling durations (0, 2, 3, 4, 5, 6, 7, and 8 weeks). After that, seventy rhizomes from each cooling duration were placed in a growing room at a temperature of 17 °C, under a 16/8 h photoperiod of 60 µmol m^−2^ s^−1^ (cool-white fluorescent lamps) to determine dormancy breaking and growth of leaves. The control plants (dormant rhizomes) grew continuously in the growing room at 17 °C. Sprouting was determined when the bud tip was slightly opened, or one leaf had emerged. The number of sprouting rhizomes was determined 2, 3, and 4 weeks after each cooling duration. After 4 weeks of growing in inducing conditions, the length of petioles and leaf number were also evaluated.

For each cooling duration, 20 buds were selected randomly and pooled to determine the contents of soluble sugars, starch, total phenolics, endogenous hormone, and gene expression.

### 4.4. Measurements of Soluble Sugar Contents

After homogenization, bud samples (approx. 100 mg) were extracted with 1.5 mL of 80% aqueous ethanol, then centrifuged at 833× *g* for 10 min. The amounts of total soluble sugars were estimated by the phenol–sulfuric method [65]. The supernatant was mixed with 5% phenol and 96% sulfuric acid. The absorbance (λ = 490 nm) of the samples was measured spectrophotometrically (Thermo Electron Corporation (Waltham, MA, USA), type Evolution 300 BB). The amounts of soluble sugars were determined against a glucose standard and expressed in grams per 100 g of fresh mass (FM) of plant tissue.

### 4.5. Measurements of Starch Content

Starch was determined in pellets remaining after soluble sugars analysis using Megazyme Total Starch Assay Kit (Neogen, Lansing, MI, USA). The pellets were rinsed with ethanol, and then 3 mL of thermostable alpha-amylase solution (1/30; alpha-amylase/sodium acetate buffer, pH 5.0) was added. The samples were vortexed and placed in a boiling water bath for 12 min. The samples were allowed to cool before 100 µL of amyloglucosidase solution was added, and the samples were placed in a 50 °C water bath for 30 min. The supernatant was mixed with glucose determination reagent (GOPOD Reagent, Neogen, Lansing, MI, USA) and incubated at 50 °C for 20 min. The absorbance (λ = 510 nm) of the samples was measured spectrophotometrically (Thermo Electron Corporation (Waltham, MA, USA), type Evolution 300 BB). The percentage of starch was directly calculated following the specific Megazyme equation based on the measured absorbance values.

### 4.6. Measurements of Total Phenolic Contents

To estimate the phenolic content, approx. 100 mg of plant tissue was homogenized in 1.5 mL of 80% ethanol and centrifuged at 2800 rpm for 20 min. The supernatant was mixed with 20% Na_2_CO_3_ and Folin–Ciocalteu reagent [66]. The absorbance (λ = 760 nm) of samples was estimated spectrophotometrically (Thermo Scientific Evolution 300, Waltham, MA, USA). The total phenolic content was calculated as milligrams of chlorogenic acid per gram of FM of plant tissue.

### 4.7. Quantification of Aux, ABA and SA

The rhubarb buds were frozen in liquid nitrogen immediately after collection, and then they were lyophilized and homogenized while still frozen. Then, 50 mg of pulverized plant material was used for each sample. Phytohormones were extracted with a 1 mL mixture of methanol/water/formic acid (15/4/1; *v*/*v*/*v*) according to Dobrev and Kaminek [67] with modifications by Stefancic et al. [68]. An internal isotopic standard mixture consisting of deuterated IAA, SA, and ABA was added to each sample. The extract was then centrifuged, the supernatant was collected, and the extraction procedure was repeated. The combined supernatant was dried and reconstituted in 1 mL 1 M formic acid. This extract was fractionated with SPE columns Oasis MCX 1cc/30 mg (Waters, Milford, MA, USA).

The acidic fraction was eluted from the SPE column with 1 mL methanol, evaporated to dryness, and reconstituted in 50 μL methanol. Samples prepared in this manner were analysed on HPLC column Supelco Ascentis RP-Amide (Saint Louis, MO, USA) (7.5 cm, 4.6 mm, 2.7 μm). Mobile phases were 0.1% formic acid solution in water (solvent A) and acetonitrile/methanol (1/1) mixture. Gradient elution was applied under the flow rate of 0.5 mL/min. HPLC apparatus was Agilent Technologies 1260 equipped with Agilent Technologies 6410 Triple Quad LC/ MS with ESI (Electrospray Interface, Agilent Technologies, Santa Clara, CA, USA). The two most abundant secondary ions were monitored (MRM—multiple reaction monitoring modes) for the most analysed compounds. One was used for quantification (quantifier ion), whereas the second was used for additional identity confirmation (qualifier ion). The monitored ions were: indole-3-acetic acid (IAA)—*m*/*z* 176.1 primary, 130.3, 77.2 secondary; indolebutyric acid (IBA)—*m*/*z* 204.1 primary, 186.4, 130.3 secondary; D-IAA (deuterated IAA used as internal standard)—*m*/*z* 181.1 primary, 134.7 secondary; salicylic acid (SA)—*m*/*z* 139.0 primary, *m*/*z* 121.0 and 39.0 secondary; D-SA (deuterated SA used as internal standard)—*m*/*z* 143.1 primary, *m*/*z* 125.1 secondary; abscisic acid (ABA)—*m*/*z* 265.2 primary, *m*/*z* 229.1, 247.1 secondary; D-ABA (deuterated ABA used as internal standard)—*m*/*z* 271.2 primary, *m*/*z* 167.1 secondary. Ten-point calibration curves were prepared for the analyzed compounds.

### 4.8. Molecular Analysis

Molecular studies included the expression analysis of 12 genes related to carbohydrate, flavonoids and phytohormone metabolism and signal transduction pathways. To examine the expression of genes, RNA was extracted according to Chang et al. [69]. DNA traces were removed from RNA samples by digestion with RQ RNase-Free DNase (Promega, Madison, WI, USA), and then RNA samples were purified using RNeasy Mini Kit (Qiagen, Hilden, Germany) according to the protocol for RNA clean-up. Concentration and purity of the total RNA were examined using an Epoch spectrophotometer (BioTek, Highland Park, VT, USA) in duplicate. From each sample, 1 µg of RNA was reverse-transcribed using M-MLV reverse transcriptase (Promega, Madison, WI, USA) and oligo (dT)_15_ primer (Promega, Madison, WI, USA) in a 25 µL reaction volume. Obtained cDNA samples were used for the gene expression analysis performed using the quantitative real-time PCR (qRT-PCR) technique with specific primers [8,69]. Relative expression was based on expression of the *GAPDH* (*glyceraldehyde-3-phosphate dehydrogenase*) gene which was applied as a reference gene [70]. Quantitative RT-PCR was carried out in LightCycler^®^480 machine (Roche, Basel, Switzerland) using KAPA^TM^ SybrFast qPCR Master Mix (Kapa Biosystems, Amsterdam, The Netherlands), according to manufacturer’s instructions, in a total volume of 20 µL and 1/10 cDNA dilution for each tested sample. The annealing temperature for all primers was 58–60 °C, depending on primers. Four ten-fold dilutions of cDNA were run together with analyzed samples for a calculation of the standard curve (correlation coefficient >0.99) and the PCR efficiency. The relative quantification of the mRNA level of tested genes was read out from the standard curve and normalized to the reference gene. Fold change was calculated using the standard 2^−^^ΔΔCT^ method.

### 4.9. Statistical Analysis

All the gene expression data were analyzed by using the LighCycler^®^480 software v. 1.5.0. (Roche, Basel, Switzerland). Quantitative RT-PCR data represented the medium of at least two independent biological replications with each done using the three technical repetitions. Standard deviation represents the variation between the biological repetitions. Microsoft Office 365 (Redmond, WA, USA) was used for figure construction.

## 5. Conclusions

Morphological changes, such as the growth cessation, and yellowing and dying of leaves reflect the process of para- and endodormancy development in micropropagated rhubarb ‘Malinowy’ plantlets under the high temperature. This study indicates a significant increase in the starch, total phenolics, ABA, IAA and SA levels in the buds during transition from para- to endodormancy. The rhizomes cooled at 4 °C for eight weeks showed the largest sprouting rate in the shorter time period and produce the longest stalks rhubarb ‘Malinowy’ than those cooled for five to seven weeks. These results provide the first indication of carbohydrate metabolism, plant hormone synthesis and signal transduction in underground buds of rhubarb associated with dormancy release. The relationship between phenolics, including SA levels and dormancy status was also observed. Understanding the mechanisms of dormancy regulation of rhubarb may be essential for the development of successful techniques for rhubarb forcing and off-season production of planting material.

## Figures and Tables

**Table 1:** Growth and senescence of rhubarb ‘Malinowy’ plantlets propagated in vitro after different growth periods in the greenhouse. Means indicated with the same letter within each growth parameter do not differ significantly (*p* = 0.05) according to Duncan’s test.

Growth Duration	Length of Leaf Petioles (cm)	Leaf Area (cm^2^)	Yellow Leaves (%)	Rhizome Mass (g)
1 month	7.27 a	28.74 a	-	0.883 a
2 months	10.25 b	74.75 b	-	2.200 b
3 months	9.63 b	74.21 b	29.6	4.325 c
4 months	-	-	79.7	6.574 d

## Data Availability

The data presented in this study are available on request from the corresponding author.
